# Asymmetric phospholipid: lipopolysaccharide bilayers; a Gram-negative bacterial outer membrane mimic

**DOI:** 10.1098/rsif.2013.0810

**Published:** 2013-12-06

**Authors:** Luke A. Clifton, Maximilian W. A. Skoda, Emma L. Daulton, Arwel V. Hughes, Anton P. Le Brun, Jeremy H. Lakey, Stephen A. Holt

**Affiliations:** 1 ISIS Pulsed Neutron and Muon Source, Science and Technology Facilities Council, Rutherford Appleton Laboratory, Harwell, Oxfordshire OX11 OQX, UK; 2 Department of Chemistry, University of Bath, Bath BA2 7AY, UK; 3 Bragg Institute, Australian Nuclear Science and Technology Organisation, Locked Bag 2001, Kirrawee DC, New South Wales 2232, Australia; 4 Institute for Cell and Molecular Biosciences, Newcastle University, Framlington Place, Newcastle upon Tyne NE2 4HH, UK

**Keywords:** lipopolysaccharide, Gram-negative bacterial outer membrane, neutron reflection, rough mutant lipopolysaccharides, isotopic labelling

## Abstract

The Gram-negative bacterial outer membrane (OM) is a complex and highly asymmetric biological barrier but the small size of bacteria has hindered advances in *in vivo* examination of membrane dynamics. Thus, model OMs, amenable to physical study, are important sources of data. Here, we present data from asymmetric bilayers which emulate the OM and are formed by a simple two-step approach. The bilayers were deposited on an SiO_2_ surface by Langmuir–Blodgett deposition of phosphatidylcholine as the inner leaflet and, via Langmuir–Schaefer deposition, an outer leaflet of either Lipid A or *Escherichia coli* rough lipopolysaccharides (LPS). The membranes were examined using neutron reflectometry (NR) to examine the coverage and mixing of lipids between the bilayer leaflets. NR data showed that in all cases, the initial deposition asymmetry was mostly maintained for more than 16 h. This stability enabled the sizes of the headgroups and bilayer roughness of 1,2-dipalmitoyl-*sn*-glycero-3-phosphocholine and Lipid A, Rc-LPS and Ra-LPS to be clearly resolved. The results show that rough LPS can be manipulated like phospholipids and used to fabricate advanced asymmetric bacterial membrane models using well-known bilayer deposition techniques. Such models will enable OM dynamics and interactions to be studied under *in vivo*-like conditions.

## 1. Introduction

Bacteria are differentiated into two main groups, Gram-positive or Gram-negative, based on a technique which detects the thick peptidoglycan cell wall characteristic of Gram-positive bacteria. Gram-negative bacteria are of particular biomedical, technological interest owing to their increasing antibiotic resistance and their utility in many biotechnological processes. The most commonly known example is *Escherichia coli*, found naturally in our digestive system and extensively used in biomedical research and industry. However, some strains may cause food poisoning, septicaemia or meningitis while, in developing countries, it remains a major cause of infant mortality. Furthermore, this group also includes *Klebsiella* (hospital-acquired infections), *Legionella* (Legionnaires' disease), *Neisseria* (meningitis and gonorrhoea), *Pseudomonas* (lung infections in cystic fibrosis patients) and even *Yersinia pestis* (bubonic plague). However, just as *Legionella* was unknown until recently, previously unnoticed Gram-negative bacteria such as *Acinetobacter* are now a significant threat to hospital patients and are rapidly acquiring multiple antibiotic resistances. As the outer membrane (OM) presents an additional barrier to antibiotics entering Gram-negative bacteria, biophysical and structural studies are of significant interest [1].

The Gram-negative OM resembles most biological membranes being a lipid bilayer with embedded membrane proteins, however it is extremely asymmetric [2]. In lipid terms, the inner, cytoplasmic, membrane of Gram-negative bacteria is composed predominately of phospholipids, in particular phosphatidylethanolamine and phosphatidylglycerol, as well as cardiolipin [3]. The OM has a phospholipid-rich inner leaflet, with a similar composition to the cytoplasmic membrane however, the outer leaflet which faces the extracellular environment, is predominantly composed of lipopolysaccharides (LPSs) [2].

LPSs are complex molecules which can be considered to consist of three parts. Lipid A, which is a phosphorylated diglucosamine (di-GlcN) molecule with covalently attached acyl chains, which anchors the LPS molecule to the hydrophobic interior of the OM. Attached to the glucosamine (GLcN) headgroup of Lipid A and facing the outer surface is the core oligosaccharide region, which can be further broken down into the inner and outer core. The inner core is composed of the sugars 3-deoxy-d-manno-octulsonic acid (Kdo) and l-glycero-d-manno-heptose (Hep) and the outer core region is composed of sugars such as hexoses and hexosamines. Attached to the core is the O-antigen region, the largest part of LPS and composed of a repeating chain of oligosaccharides with high variability across bacterial strains [4,5]. The charge on the Gram-negative bacterial OM (GNB-OM) surface is negative owing to the high levels of phosphorylation of both the GLcNs on Lipid A and the Kdo and Hep groups in the inner core [5].

In Gram-negative bacteria, LPS may be present in the smooth or rough form. Smooth LPS contains the complete core oligosaccharide and O-antigen regions. Bacterial colonies which possess these LPS types form visibly smooth colonies on agar plates, hence the name. Colonies of bacteria expressing types of LPS which do not contain the O-antigen region with either complete or truncated core oligosaccharide regions appear roughened and are termed rough mutants [6]. Rough mutant LPSs are obtained from mutated bacteria which are, in general, not found in nature but are viable, with the genes which encode for LPS formation altered to produce a truncated LPS in the OM outer leaflet [7].

Previous studies have examined the structure of model Gram-negative bacterial membranes composed of deep rough, rough and smooth LPS in bilayer structures composed of LPS only or LPS/phospholipid mixtures. Studies have ranged from examining the formation, structure and physiochemical properties of LPS-containing vesicles in solution [8,9] and in dry and hydrated powders [10]. Neutron and X-ray diffraction studies have been used to examine suspensions and stacked bilayers of both smooth and rough LPS types [11,12]. Studies on deep rough LPSs in monolayers at the air–liquid interface have revealed the effect of divalent cations on the packing and interaction of antimicrobial peptides with these interfacial films [13], and recently it was shown that Rc-LPS which possesses a significant portion of the LPS core oligosaccharide region could be deposited at the air–liquid interface as stable monolayers [14]. Schneck *et al.* [15] were able to deposit smooth LPS monolayers onto a hydrophobically modified silicon surface, using these monolayers to examine the effect of Ca^2+^ on the conformation of the O-antigen.

Here, we have created and examined model Gram-negative bacterial membranes similar to the GNB-OM in both lipid components and asymmetry. These GNB-OM mimics were single bilayers deposited on the surface of silicon crystals. The GNB-OM has a phosphatidylethanolamine-rich inner leaflet and to mimic this zwitterionic phospholipid 1,2-dipalmitoyl-*sn*-glycero-3-phosphocholine (DPPC) was deposited as the first layer [2,3]. The LPS outer leaflet was composed of either *E. coli* Lipid A, or the rough mutant LPSs, Rc-LPS or Ra-LPS.

**Figure 1.**
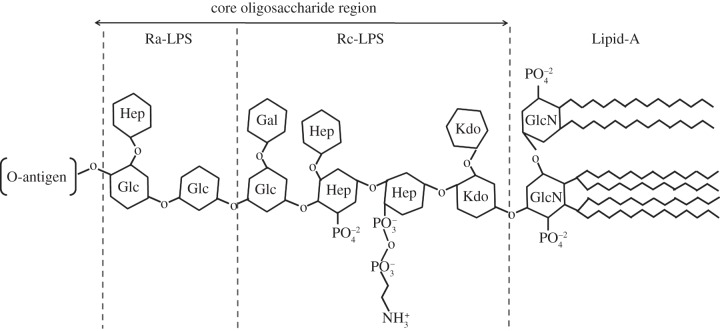
A cartoon representation of the structure of *E. coli* lipopolysaccharide, showing the lipid tails and sugar groups. The limiting regions corresponding to Lipid A and Rc and Ra-LPS from rough mutant bacterial strains are shown. Within this general model there are small variations of the core region sugars and phosphates [5].

*Escherichia coli* Lipid A is the smallest LPS used in these studies and contains six saturated acyl chains attached to a GlcN headgroup [5]. In addition to Lipid A, *E. coli* Rc-LPS contains a significant proportion of the Hep, glucose (Glc), galactose (Gal) and Kdo of the LPS core oligosaccharide region, whereas Ra-LPS contains the complete inner core region [10] (figure 1).

Neutron reflectometry (NR) was used to examine the structure normal to the interface of asymmetric bilayers deposited on the silicon surface. This study examines the structural asymmetry and stability of three differing model OMs with increasing core oligosaccharide size, and therefore increasing compositional similarity to smooth GNB-OM.

The present systems biology investigation provides a holistic approach for the detailed study of realistic models of the GNB-OM ranging from the synthesis and assembly on a well-defined surface to the precise quantitative determination of structure and composition via multi-contrast fitting of NR data. Our realistic model was relatively easily obtained and owing to this it can serve as a platform for more advanced studies of the GNB-OM at the molecular level, such as interaction/binding studies, transport, complexation, kinetic studies to name a few.

## 2. Material and methods

### 2.1. Materials

Lipid A (diphosphoryl from *E. coli* F583), Rc mutant rough strain LPS (Rc-LPS, from J5 *E. coli*) and Ra mutant rough strain LPS (Ra-LPS, from EH100 *E. coli*) were obtained from Sigma-Aldrich (Dorset, UK). DPPC and tail-deuterated DPPC (d-DPPC, 1,2-dipalmitoyl(d62)-sn-glycero-3-phosphocholine) were obtained from Avanti polar lipids (Alabaster, AL, USA). All phospholipid and LPS samples were used without further purification. All other chemicals were sourced from Sigma-Aldrich.

### 2.2. Asymmetric bilayer deposition

Model Gram-negative bacterial membranes were deposited on the Piranha-cleaned (SiO_2_) surface of single silicon crystals using a purpose-built Langmuir–Blodgett (LB) trough (KSV-Nima, Biolin Scientific, Finland) [16]. LB deposition was used to deposit the inner leaflet of the membrane on the silicon surface and Langmuir–Schaefer (LS) deposition used for the outer leaflet [17] (for a pictorial description, see the electronic supplementary material, figure S1). For the LB deposition of the inner bilayer leaflet, tail-hydrogenated DPPC (h-DPPC) or d-DPPC was deposited from chloroform onto a clean air–liquid interface of non-buffered water and compressed to a surface pressure of 27 mN m^−1^. A submerged silicon crystal was then lifted through the air–water interface at a speed of 3 mm min^−1^ while surface pressure was kept constant. The LB trough was then cleaned and an air/liquid interfacial monolayer of Lipid A, Rc-LPS or Ra-LPS was deposited on the water surface (from 60% CH_3_Cl, 39% MeOH and 1% H_2_O v/v) [14] and compressed to 27 mN m^−1^. The LS deposition of the bilayer outer leaflet was achieved by placing the silicon crystal containing the LB-deposited DPPC monolayer in a holder directly above the air–liquid interface of the LB trough. The angle of crystal adjusted using a purpose-built levelling device to make crystal face parallel to the water surface. The silicon crystal (and LB film) was then dipped through the interface at a constant speed of 3 mm min^−1^ and lowered into a purpose-built sample cell in the well of the trough. All bilayer deposition took place under ambient conditions and without subphase buffering until NR analysis.

Initially, 27 mN m^−1^ was chosen as the monolayer deposition pressure for the fabrication of the bilayers as DPPC is in the condense phase at this surface pressure under the ambient conditions [18,19]. It was discovered that high coverage bilayers of asymmetrically deposited bilayer of DPPC (inner leaflet) and Lipid A (outer leaflet) (*DPPC* : *Lipid A*) could be deposited with both the inner and outer bilayer leaflets deposited at 27 mN m^−1^ (see results section). Therefore, this pressure was then used for the deposition of all bilayer samples described here.

### 2.3. Neutron reflectometry measurements

Specular NR measurements were carried out using the INTER [20], SURF [21] and CRISP [22] time-of-flight reflectometers at the Rutherford Appleton Laboratory (Oxfordshire, UK), using neutron wavelengths from 0.5 to 6.5 Å for CRISP, 0.5 to 6.8 Å for SURF and 1 to 16 Å for INTER. The reflected intensity is measured as a function of the momentum transfer, *Q*_z_ (*Q*_z_ = (4*π* sin *θ*)/*λ*, where *λ* is wavelength and *θ* is the incident angle). The collimated neutron beam was reflected from the silicon–liquid interface at different glancing angles of incidence, being 0.35°, 0.8° and 1.8° (for CRISP), 0.35°, 0.65° and 1.5° (for SURF) and 0.7° and 2.3° (for INTER).

Purpose-built liquid flow cells for analysis of the silicon–liquid interface were placed on a variable angle sample stage in the NR instrument and the inlet to the liquid cell was connected to a liquid chromatography pump (L7100 HPLC pump, Merck, Hitachi), which allowed for easy exchange of the solution isotopic contrast within the (3 ml volume) solid–liquid sample cell. For each solution isotopic contrast change, a total of 22.5 ml of 20 mM pH/D 7.0 sodium phosphate buffer solution was pumped through the cell (7.5 cell volumes) at a speed of 1.5 ml min^−1^. This was found by examination of the NR data to completely exchange the solution in the cell from one isotopic contrast to another. Each solution contrast was run in duplicate with the repeat analysis taken at 16 h intervals. This was conducted to check the stability of the bilayer over time and under periodic flow (due to changing the solution contrast within the solid–liquid flow cell).

### 2.4. Neutron reflectometry data analysis

Reflectivity profiles were obtained from series of samples, which were chemically similar but differed in the isotopic (deuterium) composition of either aqueous or lipid contents. Specifically, the isotopic contrast series contained data from two bilayers which differed in phospholipid isotopic contrast labelling (one h-DPPC labelled and another d-DPPC labelled) which were measured under three-solution isotopic contrasts yielding a total of six different reflectivity profiles for each model membrane.

As there is no isotopic contrast between the tails of the hydrogenated phospholipid and the hydrogenated LPS (table 1), there is no way of determining the contribution of each individual component to the bilayer structure if only hydrogenous components are examined. However, owing to the large difference in neutron scattering length density (SLD, *ρ*) between hydrogenated and deuterated alkyl chains the use of deuterated and hydrogenated lipids within the same bilayer can highlight asymmetry in the inner and outer leaflet composition and allow for the structural parameters from the lipid tails in individual bilayer leaflets to be determined [26]. As DPPC is relatively easily obtained in its deuterium labelled form from a commercial supplier, this was used as the deuterated lipid component in the work described here. The LPS was hydrogenous (i.e. natural abundance) material. Table 1 gives a list of the neutron SLD of the components used in this study.

**Table 1.** Summary of scattering length densities of the lipid components studied and the solution subphases.

lipid/solvent	^a^neutron scattering length density (*ρ*) (×10^−6^ Å^−2^)
20 mM pD 7.0 D_2_O phosphate buffer	6.35
20 mM pH/D 7.0 SMW phosphate buffer	2.07
20 mM pH 7.0 H_2_O phosphate buffer	−0.56
silicon	2.07
silicon oxide (SiO_2_)	3.41
DPPC headgroup	1.98
h-DPPC tails	−0.39
d-DPPC tails	7.45
Lipid A tails	−0.39
Lipid A GlcN (headgroup) in D_2_O	3.39
Lipid A GlcN (headgroup) in H_2_O	2.58
Rc-LPS hydrophilic core oligosaccharide (headgroup) region in D_2_O	4.2
Rc-LPS hydrophilic core oligosaccharide (headgroup) region in H_2_O	2.04
Ra-LPS hydrophilic core oligosaccharide (headgroup) region in D_2_O	4.28
Ra-LPS hydrophilic core oligosaccharide (headgroup) in H_2_O	2.01

^a^The volumes used to calculate SLD for Lipid A and LPS headgroups are based on volumes from the crystal structures of sugars [14,23]. Values from H_2_O, D_2_O, Si and SiO_2_ have been reported previously [24,25].

The DPPC : LPS bilayers were examined under three-solution isotopic contrast conditions which were used to highlight the different components of the bilayer structure. Reflectivity profiles were obtained with a solution subphase of D_2_O (99.9%, *ρ* of 6.35 × 10^−6^ Å^−2^), silicon scattering length density matched water (SMW, 38% D_2_O: 62% H_2_O v/v; *ρ* = 2.07 × 10^−6^ Å^−2^) and water (*ρ* = −0.56 × 10^−6^ Å^−2^).

Neutron reflectivity profiles were simultaneously analysed using RasCal [27], which employs an optical matrix formalism (described in detail by Born & Wolf [28]) to fit layer models to the interfacial structure. In this approach, the interface is described as a series of slabs, each of which is characterized by its SLD, thickness and roughness. The reflectivity for the model starting point is then calculated and compared with the experimental data. A least-squares minimization is used to adjust the fit parameters to reduce the differences between the model reflectivity and the data. In all cases, the simplest possible model (i.e. least number of parameters (layers)), which adequately described the data, was selected. NR profiles obtained from samples under differing solution isotopic conditions were constrained to fit to the same layer and thickness profile with SLD varied between datasets as required.

The fitted results of reflectivity data obtained from d-DPPC-labelled bilayers (in particular the tail layer SLDs) at three differing solution H_2_O/D_2_O mixtures (100% H_2_O, 38% D_2_O and 100% D_2_O) were used to determine the relative contribution of the three membrane components, DPPC, LPS and water, to the inner leaflet (closest to the silicon surface) and outer leaflet (furthest from the Si surface) tails of the bilayer using a set of linear equations. The three individual components of a fitted layer within the bilayer will contribute to the SLD of this layer as shown in equation (2.1)2.1
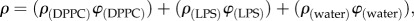
where *ρ* is the SLD of a given layer. *ρ*_(DPPC)_, *ρ*_(LPS)_ and *ρ*_(water)_ are the individual SLDs of the DPPC, LPS and solvent, respectively (values are given for these in table 1) and *φ*_(DPPC)_, *φ*_(LPS)_ and *φ*_(water)_ are the volume fractions of these components within a particular layer. In the tail regions of the bilayers, *φ*_(water)_ can be determined by the difference in *ρ* of the lipid tail layers in H_2_O, D_2_O and SMW solvent contrasts, which will be owing to the water contribution to this region of the bilayer only as the DPPC and LPS lipid tails do not possess labile hydrogens, and therefore will not undergo solvent-contrast-related changes in SLD [16]. *φ*_(water)_ was determined by2.2
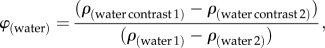
where *ρ*_water contrast 1_ and *ρ*_water contrast 2_ are the SLDs of the same tail layer in two different water isotopic contrasts (in this case either D_2_O, SMW or H_2_O) and *ρ*_water 1_ and *ρ*_water 2_ are the SLDs of each H_2_O/D_2_O mix, respectively. In this way, *φ*_(water)_ and *ρ* − (*ρ*_(water)_*φ*_(water)_) are obtained, these values relate the relative contributions of DPPC and LPS tails to this layer by2.3
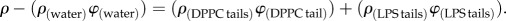


Therefore, once *φ*_(water)_ and *ρ* − (*ρ*_(water)_ *φ*_(water)_) were known, these values were used to determine the relative mixing of the hydrogenated LPS and the deuterated phospholipid in the bilayer leaflets. The *φ*_(DPPC tails)_ in the tail layers of the bilayer was determined by2.4
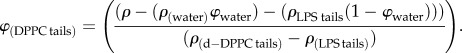


Once *φ*_(DPPC tails)_ was determined, the contribution of *φ*_(LPS tails)_ to these layers was deduced by2.5
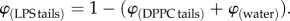


The relative volume fractions of the LPS and DPPC in the headgroup layers of the bilayer structures were not able to be determined owing to the minimal isotopic contrast between the DPPC headgroups and the LPS core oligosaccharide region (table 1). Therefore, all volume fractions of DPPC, LPS and water quoted in this article are describing the lipid tail regions of each leaflet within the bilayer.

### 2.5. Model to experimental data fitting error analysis

Model to experimental data fitting errors were obtained using Rascals ‘bootstrap’ error analysis function, in which the original dataset is resampled and these new datasets fitted via the same methods as described earlier. The parameter value distributions obtained across these fits were used to estimate errors which were then propagated through the calculations of the derived parameters according to standard error treatment methods [29].

## 3. Results

### 3.1. Asymmetric *DPPC* : *Lipid A* bilayer

**Figure 2.**
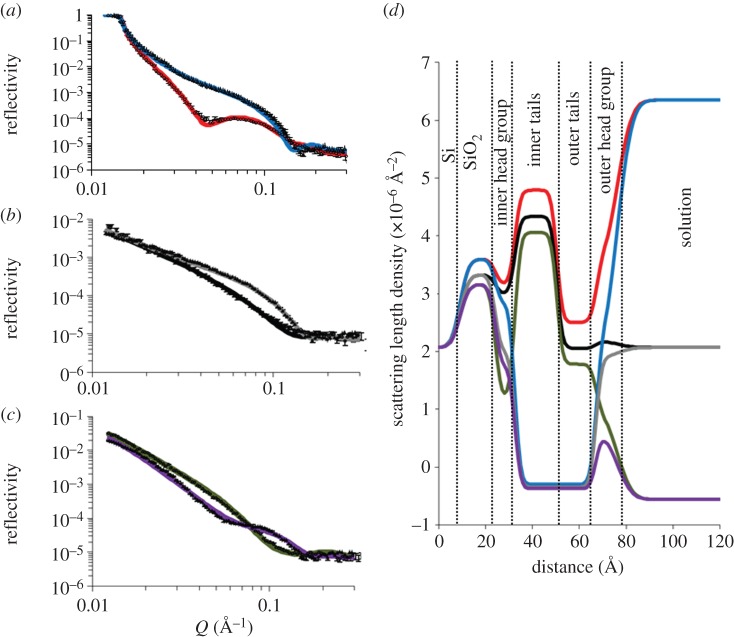
Neutron reflectometry profile and model data fits (*a*–*c*) and the scattering length density profiles these fits describe (*d*) for asymmetrically deposited DPPC (inner leaflet) : Lipid A (outer leaflet) bilayer. The six simultaneously fitted isotopic contrasts shown are (*a*) *d*-*DPPC* : *Lipid A* in D_2_O (red line), *h*-*DPPC* : *Lipid A* in D_2_O (blue line); (*b*) *d*-*DPPC* : *Lipid A* in SMW (black line), *h*-*DPPC* : *Lipid A* in SMW (grey line); (*c*) *d*-*DPPC* : *Lipid A* in H_2_O (green line), *h*-*DPPC* : *Lipid A* in H_2_O (purple line). (Online version in colour.)

Figure 2 shows the NR profiles, model data fits and the resulting SLD profiles for an asymmetrically deposited *DPPC* : *Lipid A* bilayer deposited on a silicon surface. As mentioned previously, reflectivity data for two individual bilayers, an h-DPPC- and a d-DPPC-labelled bilayer, were examined under three-solution contrasts (H_2_O, SMW and D_2_O) producing six reflectivity profiles. During fitting of the data, the layer thicknesses and roughness of both bilayers were constrained to fit a single profile, however the hydration and the SLD of the layers was fitted individually for each bilayer.

The NR obtained from the *DPPC* : *Lipid A* bilayer was fitted to a five-layer model of the interfacial structure. This model represents the minimal number of layers with which the reflectivity data could be fitted. The layers in this structural model describe (moving from silicon to the bulk solution) a silicon oxide layer (1st layer), the inner bilayer leaflet headgroups (2nd layer), the inner bilayer leaflet acyl chains (3rd layer), the outer leaflet acyl chains (4th layer) and the outer leaflet headgroups (5th layer). Table 2 describes the structural parameters obtained from fitting of the NR data obtained from the d-DPPC-labelled *DPPC* : *Lipid A* bilayer sample. It should be noted that fitting of the asymmetrical *DPPC* : *Lipid A* bilayer to a simple five-layer description of the interfacial structure produced fits that were less complete than those obtained from fitting the reflectivity profiles obtained from the Rc- or Ra-LPS-containing bilayer samples using the same model.

**Table 2.** Structural parameters obtained for an asymmetrically deposited d-DPPC (inner leaflet) *E. coli* Lipid A (outer leaflet) bilayer deposited on a silicon surface at 27 mN m^−1^ monolayer pressure.

layer	thickness (Å)	*φ*_DPPC_	*φ*_Lipid A_	*φ*_water_	roughness (Å)
layer 1 silicon oxide	14.3 ± 2.9	n.a.	n.a.	0.07 ± 0.06	2.9 ± 1.3
layer 2 inner headgroup	8.5 ± 1.0	0.54 ± 0.03	0.36 ± 0.05	0.092 ± 0.050^a^	bilayer roughness = 2.4 ± 1.5
layer 3 inner tails	19.8 ± 2.0				
layer 4 outer tails	17.6 ± 3.4	0.26 ± 0.03	0.65 ± 0.06	0.15 ± 0.03^a^	as above
layer 5 outer headgroup	8 ± 5				

^a^*φ*_water_ for the headgroups does not include water of hydrations as this is accounted for in the headgroup volume fraction.

Analysis of the reflectivity data revealed high coverage for both bilayers examined (h-DPPC and d-DPPC labelled). Based on the hydration of the lipid tail regions of the bilayer, the fully hydrogenated bilayer (*h-DPPC* : *Lipid A*) was found to have an average surface coverage (determined by the addition of *φ*_LPS_ and *φ*_DPPC_ of the inner and outer leaflets combined) of 99 ± 5%, whereas the d-DPPC-labelled bilayer was found to have an average coverage of 91 ± 5%. Although similar, it is clear that repeated bilayer production produces bilayers with minor differences in coverage probably owing to random error during the bilayer fabrication process. As previously mentioned, coverages were determined from the combined volume fractions of the DPPC and LPS in the lipid tail regions of the bilayer as calculation of the headgroup volume fractions could not be accurately determined for reasons described previously. However based on the scattering length densities obtained from the headgroup layers (see the electronic supplementary material), the hydration of the lipid *DPPC* : *Lipid A* headgroup is likely to be significantly higher than that determined for the tail regions, which is expected owing to the hydrophilic nature of this moiety of the bilayer.

The d-DPPC-labelled *DPPC* : *LPS* bilayers were used to examine the asymmetry of the two lipid component bilayers. Analysis of the scattering length densities of the inner and outer bilayer tails of the *d-DPPC* : *Lipid A* bilayer reveals that although an asymmetrical structure had been produced there was mixing of the DPPC and Lipid A. Indeed, the outer leaflet of the bilayer was found to be composed of 65% Lipid A (*φ*_Lipid A_ = 0.65) and 26% DPPC, whereas conversely the inner bilayer leaflet showed almost the reverse mixing with 36% Lipid A and 55% DPPC found. As the inner leaflet of the bilayer was deposited as a pure DPPC layer and the outer leaflet was deposited as a pure Lipid A layer, this implies mixing of the two leaflets. However, the collection of repeat reflectivity data at 16 h intervals revealed that although significant mixing had occurred between the leaflets prior to initial NR analysis no further mixing between the layers occurred overtime under periodic flow (see the electronic supplementary material, figure S3 for a comparison of NR data).

Gerelli *et al.* [30] have recently examined the mixing of asymmetrically deposited phospholipid bilayers and have found that significant flipping between the inner and outer bilayer leaflets would only be expected when the bilayer components are in the liquid phase. As the bilayer structures described here were both deposited and examined at room temperature (20°C) where both the DPPC and the Lipid A components of the bilayer would be expected to be in the gel or subgel phases [31,32], flipping between the layers would not be expected. Therefore, the mixing between the layers observed here is only likely to have occurred during the LS deposition of the outer leaflet of the bilayer, in agreement with previously observed results for phospholipids [30].

### 3.2. Asymmetric *DPPC* : *Rc-LPS* bilayer

**Figure 3.**
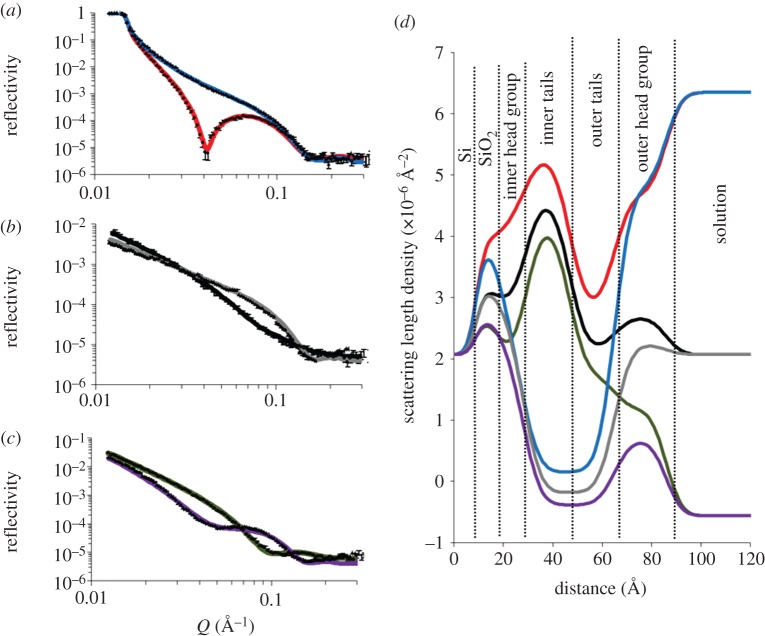
Neutron reflectometry profile and model data fits (*a*–*c*) and the scattering length density profiles these fits describe (*d*) for asymmetrically deposited DPPC (inner leaflet) : Rc-LPS (outer leaflet) bilayer. The six simultaneously fitted isotopic contrasts shown are (*a*) *d*-*DPPC* : *Rc-LPS* in D_2_O (red line), *h*-*DPPC* : *Rc-LPS* in D_2_O (blue line); (*b*) *d*-*DPPC* : *Rc-LPS* in SMW (black line), *h*-*Rc-LPS* : *Lipid A* in SMW (grey line); (*c*) *d*-*DPPC* : *Rc-LPS* in H_2_O (green line), *h*-*DPPC* : *Rc-LPS* in H_2_O (purple line). (Online version in colour.)

To examine whether more realistic mimics of the structure of the GNB-OM could be achieved, we formed bilayers with an inner leaflet of DPPC and an outer leaflet of Rc-LPS or Ra-LPS. Rc-LPS is preferable to Lipid A for use in outer bacterial membrane mimics as this rough strain LPS possesses a significant portion of the core oligosaccharide region, thus providing a better mimic of the surface structure of the GNB-OM. The use of Ra-LPS in these bilayers is better still as this rough mutant LPS contains the complete core oligosaccharide region of a full-length LPS molecule. Figure 3 shows the NR profiles, model data fits and the SLD profiles, and these fits describe for an asymmetrically deposited bilayer of DPPC (inner leaflet) and Rc-LPS (outer leaflet) (*DPPC* : *Rc-LPS*) with table 3 showing the parameters.

**Table 3.** Fitting parameters obtained for an asymmetrically deposited d-DPPC (inner leaflet) Rc-LPS (outer leaflet) bilayer deposited on a silicon surface at 27 mN m^−1^ monolayer pressure.

layer	thickness (Å)	*φ*_DPPC_	*φ*_Rc-LPS_	*φ*_water_	roughness (Å)
layer 1 silicon oxide	11.1 ± 1.9	n.a.	n.a.	0.15 ± 0.10	3 ± 2
layer 2 inner headgroup	8.4 ± 11.2	0.58 ± 0.04	0.25 ± 0.08	0.16 ± 0.06^a^	bilayer roughness = 5.4 ± 3.1
layer 3 inner tails	18.2 ± 2.5				
layer 4 outer tails	15.3 ± 3.0	0.28 ± 0.01	0.57 ± 0.02	0.15 ± 0.03^a^	as above
layer 5 outer headgroup	20.9 ± 2.0				

^a^*φ*_Water_ for the headgroups does not include water of hydrations as this is accounted for in the headgroup volume fraction.

As with the DPPC : Lipid A bilayer, a five-layer model of the interfacial structure was suitable for fitting reflectometry profiles obtained from the asymmetrically deposited *DPPC* : *Rc-LPS* bilayer. As with the DPPC : Lipid A bilayer, comparison of the coverage of the h-DPPC and d-DPPC labelled bilayers revealed minor differences in coverage between the two bilayers, with average coverages of 90 ± 10% and 84 ± 5% for the h-DPPC and d-DPPC-labelled bilayers, respectively. It should be noted however that the surface coverage for each bilayer are within error of each other.

The bilayer roughness was somewhat higher than that found for the *DPPC* : *Lipid A* membrane, fitted at 5.4 ± 3.1 Å for the Rc-LPS-containing membrane compared with 2.4 ± 1.5 Å for the Lipid A-containing membrane. The fits of the NR for d-DPPC-labelled *DPPC* : *Rc-LPS* bilayer demonstrated that the inner leaflet, which was deposited as DPPC only, contained 58 ± 4% DPPC and 25 ± 8% Rc-LPS, whereas the outer leaflet consisted of 28 ± 1% DPPC and 57 ± 2% Rc-LPS. As with the Lipid A bilayer, no change in the asymmetry was noted over time (see electronic supplementary material, figure S4).

Notably, the outer headgroup region of the *DPPC* : *Rc-LPS* bilayer was found to be significantly thicker than the equivalent region of the DPPC : Lipid A outer bilayer, being 20.9 ± 2.0 Å in thickness (compared with 8.0 ± 5.0 Å for the Lipid A-containing membrane). This difference in thickness between the two bilayers is likely owing to the presence of a significant proportion of the LPS core oligosaccharide region on the hydrophilic moiety of Rc-LPS (figure 1) compared with Lipid A.

### 3.3. Asymmetric *DPPC* : *Ra-LPS* bilayer

**Figure 4.**
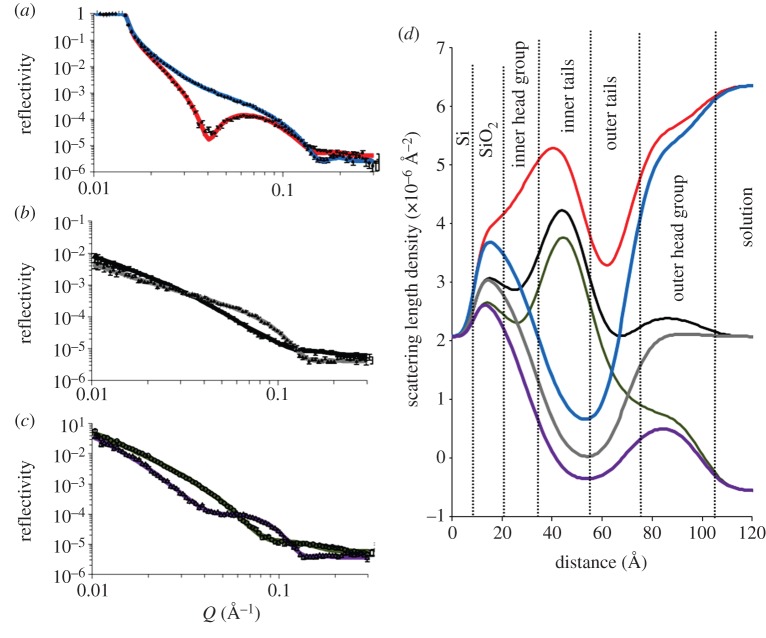
Neutron reflectometry profile and model data fits (*a*–*c*) and the scattering length density profiles these fits describe (*d*) for asymmetrically deposited DPPC (inner leaflet) : Ra-LPS (outer leaflet) bilayer. The six simultaneously fitted isotopic contrasts shown are (*a*) *d*-*DPPC* : *Ra-LPS* in D_2_O (red line), *h*-*DPPC* : *Ra-LPS* in D_2_O (blue line); (*b*) *d*-*DPPC* : *Ra-LPS* in SMW (black line), *Ra-LPS* : *Lipid A* in SMW (grey line); (*c*) *d*-*DPPC* : *Ra-LPS* in H_2_O (green line), *h*-*DPPC* : *Ra-LPS* in H_2_O (purple line). (Online version in colour.)

Ra-LPS, despite its large size and being highly water soluble, formed stable, reproducible monolayers at the air–liquid interface so that LS deposition could occur effectively (see electronic supplementary material, figure S2). The collapse pressure of the monolayer was 52 mN m^−1^, well above the 27 mN m^−1^ used in bilayer fabrication. Figure 4 shows the NR profiles, model data fits and the SLD profiles from these fits describing an asymmetrically deposited asymmetrically deposited bilayer of DPPC (inner leaflet) and Ra-LPS (outer leaflet) (*DPPC* : *Ra-LPS*) bilayer fitted to the same five-layer model structure which was previously found to be optimal for the Lipid A and Rc-LPS-containing bilayers. Table 4 lists the parameters obtained from these fits of the experimental data.

**Table 4.** Fitting parameters obtained for an asymmetrically deposited d-DPPC (inner leaflet) Ra-LPS (outer leaflet) bilayer deposited on a silicon surface at 27 mN m^−1^ monolayer pressure.

layer	thickness (Å)	*φ*_DPPC_	*φ*_Ra-LPS_	*φ*_water_	roughness (Å)
layer 1 silicon oxide	13.4 ± 2.0	n.a.	n.a.	0.104 ± 0.040	3.0 ± 1.0
layer 2 inner headgroup	14.8 ± 2.0	0.66 ± 0.05	0.19 ± 0.09	0.16 ± 0.08^a^	bilayer roughness = 7.90 ± 0.55
layer 3 inner tails	15.6 ± 0.6				
layer 4 outer tails	16.0 ± 4.8	0.22 ± 0.05	0.67 ± 0.07	0.11 ± 0.07^a^	as above
layer 5 outer headgroup	31.0 ± 1.2				

^a^*φ*_water_ for the headgroups does not include water of hydrations as this is accounted for in the headgroup volume fraction.

The total surface coverage of lipid (*φ*_DPPC_ + *φ*_Ra-LPS_) in the *DPPC* : *Ra-LPS* bilayer was found to be approximately 85% (table 4), and therefore significantly lower than that found for the DPPC : Lipid A bilayer. The structure and structural asymmetry observed across the membrane was similar to that found for the *DPPC* : *Lipid A* and *DPPC* : *Rc-LPS* bilayers, with a DPPC-rich inner leaflet (*φ*_DPPC_ = 0.66) and an LPS-rich outer leaflet (*φ*_Ra-LPS_ = 0.67), which as with the other *DPPC* : *LPS* bilayers suggested that partial asymmetry had been maintained in the interfacial film.

The most notable feature of the *DPPC* : *Ra-LPS* bilayer was the outer headgroup region of the bilayer, notably thicker (31 ± 1.2 Å) than that for Lipid A or Rc-LPS-containing membranes. This region of the bilayer structure is likely to be dominated by the contribution of the Ra-LPS headgroup region owing to the significantly larger size of the Ra-LPS hydrophillic inner core region compared with the DPPC headgroup and the higher volume fraction of Ra-LPS found in the outer bilayer leaflet tails compared with DPPC.

As previously mentioned, in all cases the bilayer structure was examined with NR over a 16 h period after initial NR measurement to asses whether any changes to the bilayer structure took place over time and after periodic flow in the solid–liquid cell. In the cases of the *DPPC* : *Lipid A*, *DPPC* : *Rc-LPS* and the *DPPC* : *Ra-LPS*, bilayer showed no significant changes to the interfacial structure over this time period (see the electronic supplementary material, figure S5).

## 4. Discussion

Here, we have examined whether it is possible to create asymmetrical GNB-OM models using Lipid A and rough mutant LPSs. The asymmetry of these GNB-OM models was intended to mimic that of the GNB-OM where a phosphatidylethanolamine-rich inner leaflet and an LPS-rich outer leaflet are found [2,5]. By varying the LPS used in the outer leaflet from Lipid A to Ra-LPS, the size of the LPS inner core region has been increased in the bilayer outer leaflet from the minimal size (GlcN only in Lipid A, figure 1) to having a complete core oligosaccharide region (Ra-LPS). In doing so, the accuracy of the membrane model moves from a bilayer that represents only the GNB-OM core hydrophobic region (*DPPC* : *Lipid A*) to a bilayer that is more structurally similar to the GNB-OM, with the Lipid A and core oligosaccharide present on the LPS in the outer leaflet of the membrane present (*DPPC* : *Ra-LPS*), and only the O-antigen missing.

Results revealed that complex asymmetrical bilayers could indeed be fabricated using both a combination of synthetic phospholipids and *E. coli* rough mutant LPSs. All the bilayers examined were found to be asymmetric in nature with a DPPC-rich inner leaflet and an LPS-rich outer leaflet. The discovery that complex amphiphillic natively extracted molecules, such as Rc- and Ra-LPS, can be incorporated into complex structures that are amenable to molecular level structural studies shows the potential of complex wild-type lipids and amphiphiles for use in the fabrication of model biological surfaces. Indeed, the GNB-OM mimics described in this study may allow for molecular level examinations of the dynamics and interactions of this membrane to be conducted under conditions close to those found *in vivo*.

Controlled deposition of LPS monolayers in multi-layered LPS only bilayer samples by LB deposition has previously been reported as intractable with LPS types possessing a core oligosaccharide region larger in size than that found in Re-LPS [10]. Here, we have been able to deposit solid supported single bilayers containing an LPS-rich outer leaflet with LPS types which contain a significant proportion of (Rc-LPS) or all of (Ra-LPS) the core oligosaccharide region. The stability of the single bilayers containing the relatively hydrophilic rough mutant LPSs may be owing to the DPPC anchoring the LPS within the bilayer. DPPC is known to form stable bilayers on an oxidized silicon crystal surface [17], with the bilayers held in place owing to a combination of electrostatic attraction between the cationic choline group on the inner bilayer leaflet and surface oxide (in this case SiO_2_) and van der Waals forces [33,34]. Hydrophobic interactions with the DPPC tails likely keep the LPS tails, and therefore the whole molecule anchored to the bilayer, which results in the stable silicon-surface-bound bilayers described here. Indeed, it has been shown previously that monolayers of smooth LPS can be deposited on to silicon surfaces hydrophobized by a covalently attached alkyl silane monolayer [15].

The structural parameters obtained for the Lipid A and LPS-rich outer leaflets of the asymmetric *DPPC* : *Lipid A* and *DPPC* : *Rc-LPS* compare well with those determined for monolayers of these lipids by Le Brun *et al.* [14]. Lipid A monolayers were found to have a headgroup thickness of 8 ± 1 Å, which compares well with the 8 ± 5 Å found for the Lipid A-rich outer leaflet of the *DPPC* : *Lipid A* bilayer. Rc-LPS monolayers at the air–liquid interface were found to have headgroup regions that had a total thickness of 29 ± 7 Å at high surface pressures, and therefore high LPS densities. We found the LPS-rich leaflet of the Rc-LPS bilayer to have a headgroup thickness of 20.9 ± 2 Å, this slightly thinner layer may suggest a tilted orientation of the Rc-LPS headgroup in the outer leaflet of the bilayer. The outer leaflet acyl chain regions of the *DPPC* : *Lipid A* and *DPPC* : *Rc-LPS* bilayers were found to be slightly thicker than found for Lipid A and Rc-LPS monolayers, which is probably owing to the presence of the palmitic acid chains of the phospholipid within this region. Previously, Snyder *et al.* [11] were able to resolve the trend of increasing core oligosaccharide region size where Re < Rd < Rc < Ra-LPS, when LPS only stacked bilayer samples were examined. Here, the same trend has been observed (Lipid A < Rc-LPS < Ra-LPS) in the complex asymmetrical single bilayer structures examined with the core oligosaccharide thicknesses (obtained from the outer leaflet headgroup thicknesses) being in general in good agreement with the aforementioned studies.

**Figure 5.**
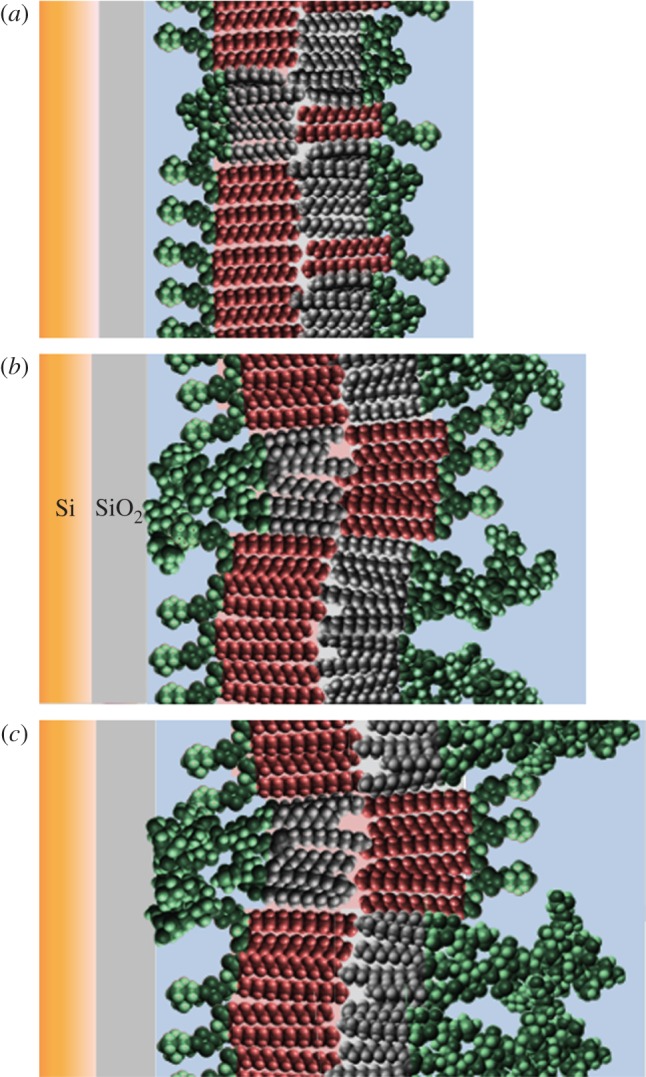
A cartoon representation of the structure across the interface found for the DPPC : LPS bilayers described here, showing the structure of (*a*) *DPPC* : *Lipid A*, (*b*) *DPPC* : *Rc-LPS* and (*c*) *DPPC* : *Ra-LPS* examined here. (Online version in colour.)

In all the bilayers examined, some mixing of the lipids between the leaflets was observed. This is likely to have occurred owing to the mechanical shock of the LS dipping phase of bilayer fabrication [30], as mixing between the leaflets was not observed over time under the ambient conditions used in these studies. The general asymmetry of the phospholipid : LPS bilayers described here showed approximately 25% LPS and approximately 65% DPPC in the inner leaflet (+10% water) and approximately 65% LPS and approximately 25% DPPC (+10% water) in the outer leaflet (figure 5 shows a cartoon representation of the interfacial structure). The GNB-OM outer leaflet is known to possess LPS predominantly as its lipid component [2], approximately 25% phospholipid found in the outer leaflet here could be considered to make these membrane models less biologically relevant. However, it should be noted that the phospholipid is a minor component of the outer leaflet. The significant asymmetry observed in these easily formed, analysed (by NR) and stable bilayer models could be considered as a reasonable representative of the GNB-OM for future biological interaction studies. Gerelli *et al*. [30] have improved phospholipid bilayer asymmetry by preparing the samples below the phase transition temperature of both lipids deposited which, for the lipids used was below room temperature. Despite the improved asymmetry when conducting the LB–LS depositions below the lipid phase transition temperatures, there was still a 10% mixing of phospholipids. The bilayers described here were all prepared at room temperature, which is well below the phase transition temperature of both the DPPC and the LPS used. Another potential way of reducing inner/outer leaflet mixing during LS deposition of the outer bilayer leaflet maybe to introduce divalent cations to the solution subphase below the LPS monolayer, as the interaction of the cations with the LPS may increase the rigidity of the monolayer causing less mixing to occur during bilayer production [13].

Total interfacial coverages of the bilayers ranged from greater than 90% for the Lipid A-containing bilayers to approximately 85% for the Rc- and Ra-LPS-containing membranes. The hydration of the tail region of the bilayers (which we use here to measure coverage) is likely owing to defects in the bilayer film, that is relatively small regions of the silicon surface with low or no lipid coverage [17], which are likely to be formed during the LS stage of the asymmetrical bilayer fabrication process. The possible reason for the slightly higher coverage for the Lipid A-containing bilayers compared with the rough LPS-containing membranes maybe owing to the higher phosphorylation found on the rough mutant LPS types. As electrostatic repulsion between neighbouring anionic LPS molecules may have caused some loss of this material during the LS deposition of the outer bilayer leaflet, the increased hydrophilicity of the rough mutant LPSs compared to Lipid A may also be causing a higher loss of the LPS to the bulk solution compared with the more hydrophobic Lipid A during the bilayer fabrication process, this may also partly account for the slightly lower coverage of the Rc- and Ra-LPS-containing bilayers.

The rough mutant LPS-containing bilayers were found to be rougher than the *DPPC* : *Lipid A* bilayer, with both the Rc- and Ra-LPS-containing films found to be 5 and 8 Å in roughness, respectively, compared with 3 Å for the *DPPC* : *Lipid A* bilayer. The size of the DPPC and Lipid A headgroup regions have been found to be the same at approximately 9 Å (table 2; [14,16]). Conversely, the headgroups of Rc- and Ra-LPS were found here to be 20.9 and 31 Å in thickness, respectively (based on the thicknesses of the LPS-rich outer leaflet headgroups; tables 3 and 4) and are thus significantly larger in size than the DPPC headgroup region. The size mismatch between the PC and LPS in the inner leaflet of the bilayer, which is next in close proximity to the relatively flat silicon oxide surface, would induce an increased roughness across the entire bilayer compared with the *DPPC* : *Lipid A* bilayer, where no significant size mismatch was present (a cartoon representation of this is shown in figure 5). Thus, the increases in roughness (and headgroup thickness in the case of the Ra-LPS-containing bilayer) are likely owing to the presence of the rough *E. coli* mutant LPS within the inner leaflet of the bilayer. The increased roughness may also be in part owing to the flexibility of the core oligosaccharide headgroup regions of Rc- and Ra-LPS [35] in the outer headgroup region making this region less defined using layer models. The interfacial roughness can be viewed as either representing a sharp interface with special variations, or as is more likely here, represents a gradual change in the neutron SLD as a function of distance. However, the interfacial roughnesses of all the bilayers studied were relatively low and were only approximately 10% of the total thickness of the membranes.

Owing to the asymmetric nature of the bilayers under study and the presence of large hydrophilic regions on Rc- and Ra-LPS-containing bilayers, the structure of the bilayers was studied over time to observe potential changes to the structure, i.e. lipid flip flop between bilayer leaflets and/or loss of material from the interface. All three bilayer types showed no change over a 16 h period in the presence of periodic fluid flows used to change isotopic contrast (see electronic supplementary material, figures S3–S5). The lack of membrane leaflet mixing observed here would be expected as both the phospholipid and LPS used in this study were examined in the gel or subgel phase [31]. It is possible that if we examined the bilayer structure above the phase transition of both DPPC and LPS which is both cases would be greater than the ambient temperature under which this study was conducted. Indeed, a relatively high temperature (more than 40°C) would be required to have both the LPS and phospholipid components in the fluid phase [31,32].

## 5. Conclusion

Bilayers which mimic lipid content and asymmetry of the GNB-OM have been successfully fabricated. The asymmetrical bilayer structures described here are composed of a mixture of synthetic phospholipids and naturally extracted LPSs making these biological membrane models accurate in the composition and asymmetry of the Gram-negative bacterial membrane which they intend to mimic. Future work will both examine interactions of the membrane models described here with antimicrobial proteins and peptides and continue to develop the complexity of the GNB-OM models; this could include embedding integral membrane proteins [36,37] and increasing the fluidity of the structures by preventing the bilayer from being in direct contact with the solid–liquid interface. However, for many methods to study Gram-negative membrane interactions, the simple, robust and long-lived model structures presented here may provide a useful tool.
